# Primary and motile cilia in the efferent ductules: a role for IFT88 in maintaining male fertility

**DOI:** 10.1007/s00018-026-06296-w

**Published:** 2026-06-23

**Authors:** Céline Augière, Louis Hermo, Shirley Ferrier-Tarin, Florence Préfontaine, Mariangela Gentile, Pietro Lupetti, Rex A. Hess, Clémence Belleannée

**Affiliations:** 1 CHU de Québec Research Center-Université Laval (CHUL), Quebec, QC Canada; 2 Centre de recherche en Reproduction, Développement et Santé Intergénérationnelle, Department of Obstetrics, Gynecology, and Reproduction, Faculty of Medicine, Université Laval, Quebec, QC Canada; 3 Department of Anatomy and Cell Biology, McGill University, Montreal, QC Canada; 4 Department of Life Sciences, University of Siena, Siena, Italy; 5 Department of Comparative Biosciences, College of Veterinary Medicine, University of Illinois at Urbana-Champaign, Urbana, IL USA

**Keywords:** Male infertility, Primary cilia, Motile cilia, Efferent ductules, IFT88, Fluid reabsorption

## Abstract

The efferent ductules are male reproductive tubules that support fertility by reabsorbing fluid and transporting sperm from testis to the epididymis. They are lined with multiciliated cells (MCCs) and reabsorptive primary ciliated cells (RPCCs). While motile cilia drive fluid flow, the contribution of primary cilia and their interaction with motile cilia remain unclear. To address this, we generated a conditional Ift88 knockout (cKO) to disrupt both primary and motile cilia in the efferent ductule epithelium. Loss of IFT88 caused shortened cilia, epithelial disorganization, and impaired fluid homeostasis. Ift88 cKO males developed early-onset infertility with luminal dilation, sperm agglutination, and obstructive azoospermia. We observed reduced ESR1 expression in proximal ductules with disruption of estrogen-dependent signaling pathways. We propose that primary cilia maintain trafficking and glycosylation machinery required for correct AQP1 localization, a key estrogen-regulated mediator of fluid reabsorption in RPCCs, as well as for postnatal epithelial maturation from a predominantly secretory to a reabsorptive state. These results identify IFT88-dependent ciliogenesis as essential for efferent ductule architecture, luminal homeostasis, and male fertility.

**Supplementary Information:**

The online version contains supplementary material available at https://doi.org/10.1007/s00018-026-06296-w.

## Introduction

**Fig. 1:**
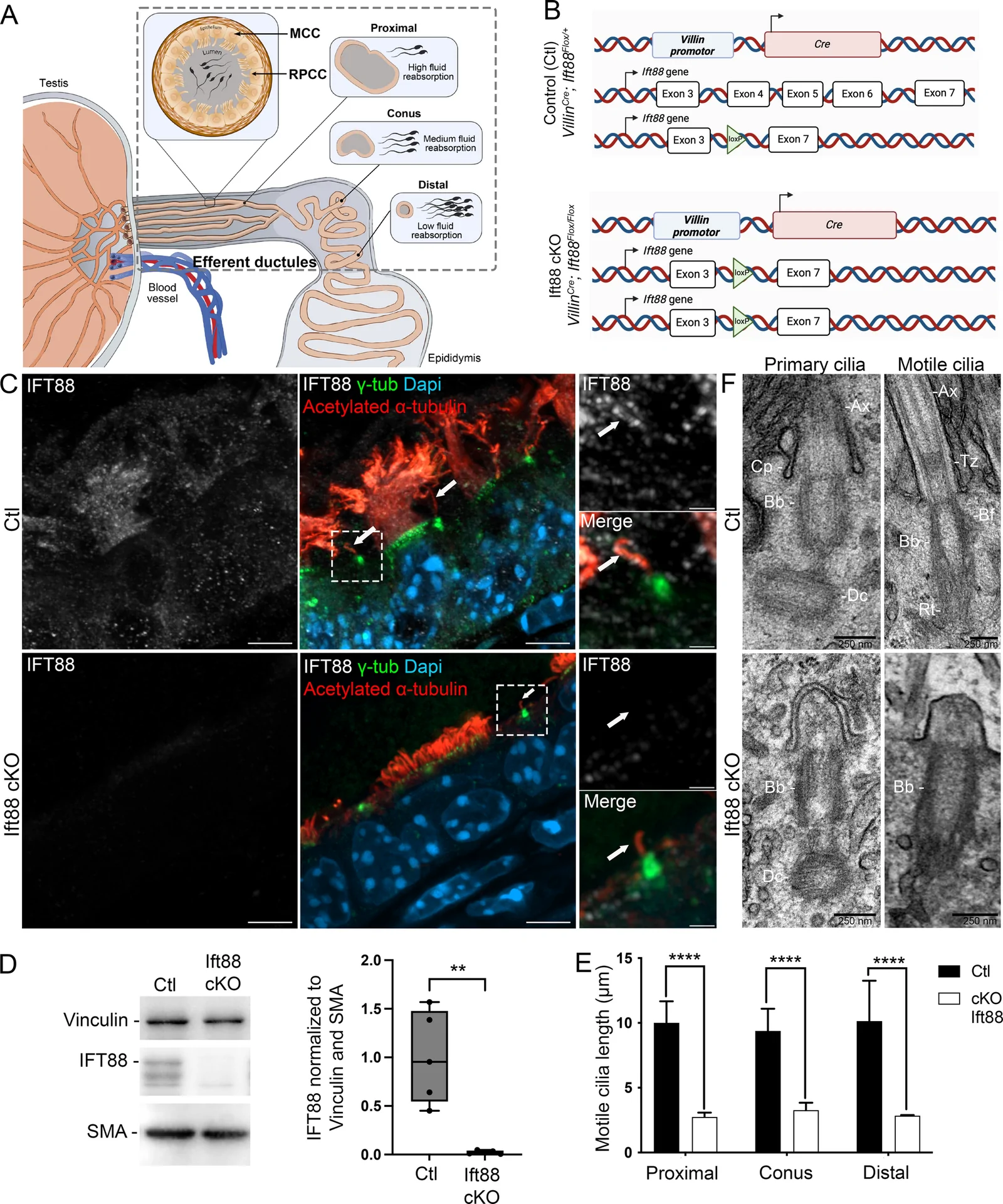
**Loss of IFT88 in* Villin-Cre *mice impairs ciliogenesis in the efferent ductules**. **A** Gross anatomy of the reabsorptive mouse efferent ductules showing the proximal, conus, and distal segments. While the proximal segment, adjacent to the rete testis, consists of convoluted tubules, the distal segment converges into a single duct connecting to the epididymis. Its epithelium is composed of two main cell types: multiciliated cells (MCC) and reabsorptive primary ciliated cell (RPCC) that ensure sperm concentration through fluid reabsorption. **B** Schematic representation of the Cre-loxP–mediated conditional knockout approach used to delete IFT88. Tissue-specific recombination was achieved through Villin-Cre-mediated excision of loxP-flanked Ift88 exons 4–6, generating *Villin*^*Cre*^; *Ift88*^*Flox/Flox*^ (Ift88 cKO) and *Villin*^*Cre*^; *Ift88*^*Flox/+*^ littermate controls (Ctl). **C** Immunofluorescent staining of efferent ductules from adult Ctl and Ift88 cKO mice showing IFT88 (gray), Ɣ-tubulin (Ɣ-tub, green) marking centrioles/basal bodies, and Acetylated ɑ-tubulin (red) labeling axoneme in primary (arrow) and motile cilia. While Acetylated ɑ-tubulin-positive cilia are observed in both Ctl and Ift88 cKO mice, IFT88-positive cilia are exclusively detected in efferent ductules from Ctl mice. Scale bar: 5 μm. Magnification of Ctl and Ift88 cKO primary cilia in dotted square are shown on right panels. Scale bar: 1 μm. **D** Western blot analysis of IFT88 in protein extracts from efferent ductules of Ctl and Ift88 cKO mice. Vinculin and Smooth Muscle Actin (SMA) served as loading control (left panel). Quantification (right panel) was performed on efferent ductule extracts from *n* = 5 mice per genotype, with values normalized to Vinculin and SMA. Data are presented as box-and-whisker plots with statistical significance determined using an unpaired t-test. **E** Motile cilia in Ift88 cKO mice are significantly shorter than in control mice across the different segments of the efferent ductules, as shown by quantitative analysis of cilia length fluorescence images. In addition, although shorter primary cilia were observed using confocal and TEM, their length could not be reliably quantified due to the frequent masking of primary cilia by dense tufts of motile cilia. Data are presented as mean ± SD and analyzed using an unpaired t-test. **p < 0.01; ****p < 0.0001. **F** Some ultrastructural components of primary and motile cilia, including the basal body (Bb), axoneme (Ax), daughter centriole (Dc), transition zone (Tz), and basal foot (Bf), were detected in the efferent ductules of control and Ift88 cKO mice using transmission electron microscopy (TEM)

Male infertility has emerged as a growing global health concern, largely driven by deficiencies in sperm function and transport within the reproductive tract, research areas that remain significantly understudied. Among the key structures of the male reproductive tract, the efferent ductules are essential for fertility [1–5], as they not only transport sperm from the testis to the epididymis but also regulate luminal fluid homeostasis, a process critical for sperm viability, function, and concentration (Fig. 1A) [6, 7].

In mice, efferent ductules (2–5 small tubules) serve to connect rete testis with the initial segment of the epididymis, and are classified as proximal, conus and distal ductules. In both humans and mice, the epithelium of the efferent ductules consists primarily of two cell types: multiciliated cells (MCC) and reabsorptive primary ciliated cells (RPCC; previously referred to as non-ciliated cells). MCCs possess only motile cilia that generate dynamic movements to propel luminal sperm [1, 8]. In contrast, RPCCs are specialized for the reabsorption of fluid and proteins from the lumen [9–11], distinguished by their microvillar border and a single primary cilium [7, 11]. While MCCs and RPCCs are recognized for their indispensable roles in governing efferent ductule physiology, important aspects of their biology remain unexplored, particularly the contribution of the RPCC’s primary cilium and the potential coordinated function with motile cilia in regulating male fertility.

Cilia are microtubule-based organelles essential for both fluid dynamics and extracellular sensory signaling [12–15]. Emerging from modified centrioles (also called basal bodies) that anchor axonemes to the membrane and the cytoskeleton, cilia are generally classified by their number, structure, and motility [16]. Motile cilia were first described in the efferent ductule epithelium in 1856 by Becker [17]. They are found in multiple numbers in MCCs, have the canonical “9 + 2” microtubule organization, and include molecules such as outer and inner dynein arms that allow for the beating motion [3, 4, 7]. In contrast to other motile ciliated epithelia, such as the trachea, ependyma, and oviduct, MCCs of the efferent ductules do not propel the luminal contents forward but rather help to mix the sperm to prevent agglutination [1–4]. Cilia beating also helps stir luminal fluids, which sustains the reabsorptive function of RPCC, as nearly 90% of the fluid arriving from the rete testis is removed, resulting in a 28-fold increase in sperm concentration [18, 19]. RPCCs are responsible for rapid isosmotic fluid reabsorption, a process tightly regulated by estrogen- and androgen-dependent ion exchangers and aquaporin channels, in addition to an elaborate endocytic/lysosomal system [5, 7, 18, 20–23]. Since the discovery of efferent ductule motile cilia in the MCCs, the presence of an additional type of cilium, the primary cilium, has also been described extending from the surface of the RPCC in different species [6, 11, 16, 24, 25], but its function in male reproductive physiology has only recently come under investigation [7]. Although motile and primary cilia share considerable basic structural and molecular similarities, they also differ significantly. For instance, the primary cilium is solitary, does not have the central paired microtubules, lacks axonemal motility proteins and is usually characterized as “9 + 0”. However at the apical surface of RPCCs, this cilium is exceptionally long with an ultrastructure that remains to be fully elucidated [6, 7].

Remarkably, the efferent ductules represent one of the very few mammalian tissues where this double ciliation exits, whereby motile and primary cilia co-exist on adjacent epithelial cells, offering a unique context to investigate their individual and cooperative roles in reproductive physiology [7, 11, 16, 24, 26, 27]. In the efferent ductules we previously demonstrated that loss of Arl13b, a component of both motile and primary cilia, resulted in shorter motile cilia (decreased ~ 25% in length), enlarged efferent ductules and partial tubular obstruction, defects attributed to impaired fluid reabsorption, which resulted in male subfertility [7]. The phenotype was relatively mild compared to that observed following complete loss of motile cilia [1–4] since Arl13b deletion did not impair ciliary beating, and the effects on RPCCs possessing primary cilia were less pronounced than those documented in models targeting fluid reabsorption like the ESR1 KO males [28–32]. These findings thus motivated us to target IFT88, a core component of the anterograde intraflagellar transport complex (IFT-B) that mediates protein transport from the basal body to the ciliary tip and is indispensable for the formation of both motile and primary cilia [33–35]. Conditional knockout of IFT88 in the efferent ductules is therefore expected to fully abrogate ciliary axoneme elongation, enabling a comprehensive assessment of cilia-dependent processes such as fluid reabsorption, tubular homeostasis, and male fertility-functions that could not be fully addressed with ARL13B loss alone.

Initially linked to polycystic kidney disease through its role in renal ciliogenesis [33], IFT88 (also known as TG737 or POLARIS) has since been shown to be essential in many tissues (for review [34]). Despite these discoveries, it has been difficult to study its role in adult tissues because complete loss of IFT88 function is incompatible with embryonic development [33]. In humans, hypomorphic mutations in IFT88 have been associated with retinal degeneration [35], but no other ciliopathy has been reported, likely because stronger mutations are incompatible with fetal survival. In the male reproductive system, a hypomorphic mutation of *Ift88* in mice was shown to allow some males to survive and reach adulthood, and interestingly these mutant mice were found to be infertile [36]. However, since that study was limited to major structural abnormalities in sperm development [36]; the potential effects of *Ift88* deletion on efferent ductule function remained unknown. To accomplish this goal, we developed a conditional knockout (cKO) mouse to selectively disrupt cilia formation in the efferent ductules without affecting other male reproductive organs. The villin-cre was selected because prior studies have shown that, in the male reproductive tract, *Villin* was expressed only in efferent ductules [7, 37]. For ciliary disruption, IFT88 was chosen, as it has already been documented in motile cilia of the efferent ductules [38].

The aim of the present study was to inhibit the growth of both primary and motile cilia, which would allow a better comparison of the resulting phenotype in cKO males to published animal models in which only motile cilia have been inhibited (Table 1). The results presented here show that IFT88 is crucial for the formation of both motile and primary cilia in the efferent ductules. Loss of IFT88 disrupts the coordinated activity of MCCs and RPCCs, leading to male infertility through luminal obstruction and impaired regulation of luminal fluid homeostasis. Mechanistically, we observed prepubertal excess fluid secretion, followed post-puberty by altered luminal fluid dynamics, proximal ductule dilation, reduced microvilli, and decreased ESR1 expression. These findings indicate a disruption of the normal establishment of postnatal luminal homeostasis during the transition to a reabsorptive state, associated with abnormal trafficking, glycosylation, and localization of the water channel aquaporin 1 (AQP1) upon conditional deletion of Ift88. This study highlights the indispensable role of ciliary assembly and function in maintaining efferent ductule function in upper regions of the male reproductive tract and points out potential new mechanisms underlying male reproductive health.

**Table 1:**
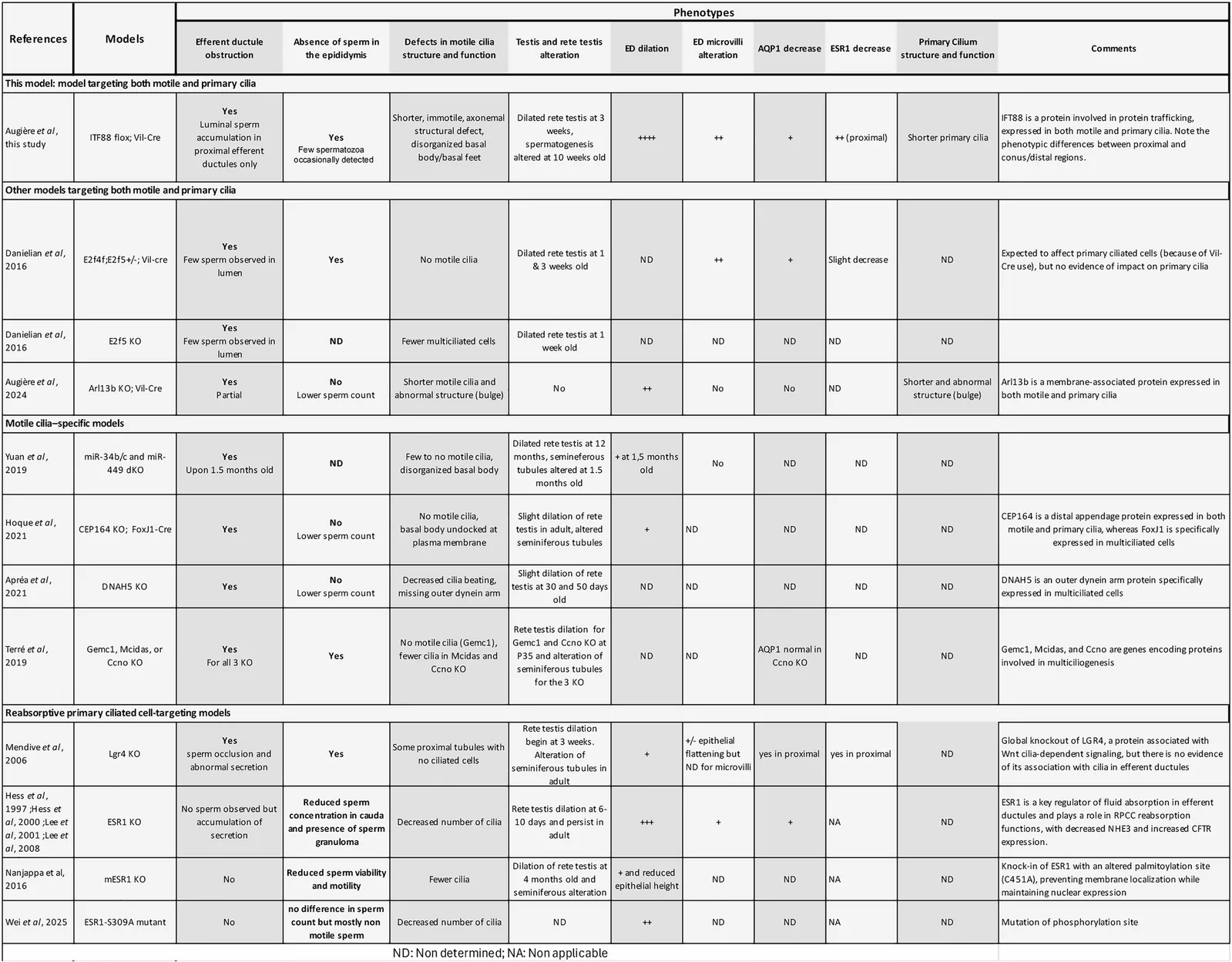
Comparative phenotypes of mouse models impairing MCC or RPCC functions in the efferent ductules

## Materials and methods

### Animals

*Ift88* conditional knock out mice were generated by crossing B6;D2‑Tg(Vil1‑Cre)20Syr mice (Mouse Models of Human Cancers Consortium, NCI‑Frederick) with *Ift88 flox/flox* mice carrying loxP sites flanking exons 4–6 (JAX #022409). In the *Vil1‑Cre; Ift88*^*flox/flox*^ (Ift88 cKO) offspring, Cre‑mediated deletion of exons 4–6 introduces a premature stop codon, thereby preventing the expression of IFT88 protein in villin-expressing efferent ducts. Control mice (Ctl) included *Vil1‑Cre; Ift88*^*flox/+*^ (hypomorphic double heterozygous) animals, along with *Ift88*^*flox/flox*^ and *Vil1‑Cre*/+ littermates. All control genotypes were experimentally evaluated and consistently displayed a normal phenotype. Primers used for genotyping are listed in Table 2. Phenotypic analyses were performed at various developmental stages, including prepuberal (3 weeks), adult (10 weeks), and aged (10 months). Following dissection, organs were weighed, and values were normalized to body weight.

**Table 2:** Primers list used in this study

Gene	Primers Forward 5’-3’	Primers Reverse 5’-3’
*Villin* ^*Cre*^	GTGTGGGACAGAGAACAAACCG	TGCGAACCTCATCACTCGTTGC
*Ift88 flox*	GAC CAC CTT TTT AGC CTC CTG	AGG GAA GGG ACT TAG GAA TGA

The Ai14 mouse (JAX stock #007914), a Cre reporter line expressing tdTomato upon recombination, was used under Villin-Cre control to assess recombination specificity and efficiency.

## Fertility test

Fertility tests were conducted on male littermates (*n* = 5 Ctl; *n* = 5 Ift88 cKO). At 8 weeks of age, each male was paired with two C57BL/6 females for 5 days. Mating was confirmed by daily monitoring for vaginal copulatory plugs. Fertility outcomes were assessed by recording the number of pups per litter.

## Tissue fixation and immunofluorescent analysis

Tissue fixation and immunofluorescent analysis were performed as described previously^7^. Briefly, the male reproductive tract was fixed in 4% paraformaldehyde and embedded in either O.C.T. compound (23-730-571, Fisher scientific) or paraffin. When required, antigen retrieval was carried out at 110 °C for 10 min using citrate buffer (10 mM sodium citrate, 0.05% Tween-20, pH 6.0). Sections were permeabilized with 1% SDS and 0.1% Triton X-100 in PBS for 4 min at room temperature, then washed in PBS. Blocking was performed in PBS containing 5% BSA and 3% goat serum for 1 h. Primary antibodies (Table 3) were diluted in DAKO solution or blocking solution and incubated overnight at 4 °C. After PBS washes, fluorophore-conjugated secondary antibodies were applied for 2 h at room temperature. For basal body and basal foot staining, efferent ducts were dissected and fixed in cold methanol for 1 h. Samples were then incubated with primary antibody against Poc1b for 2 h at room temperature, followed by a 2-hour incubation with Alexa 488-conjugated Centriolin and the appropriate secondary antibody for Poc1b. Nuclei were counterstained and slides mounted using Vectashield with DAPI (VECTH1200, MJs BioLynx). Image acquisition was performed using a Zeiss LSM900 confocal microscope with Airyscan. LAMP2 quantification was performed using standardized immunofluorescence quantification procedures where samples (*n* = 3 controls and *n* = 3 IFT88 cKO) were processed in parallel under identical experimental conditions (fixation, antibody incubation, imaging acquisition and post-acquisition settings). Quantification was then carried out by ImageJ by measuring the total fluorescence intensity normalized to cell area.

**Table 3:**
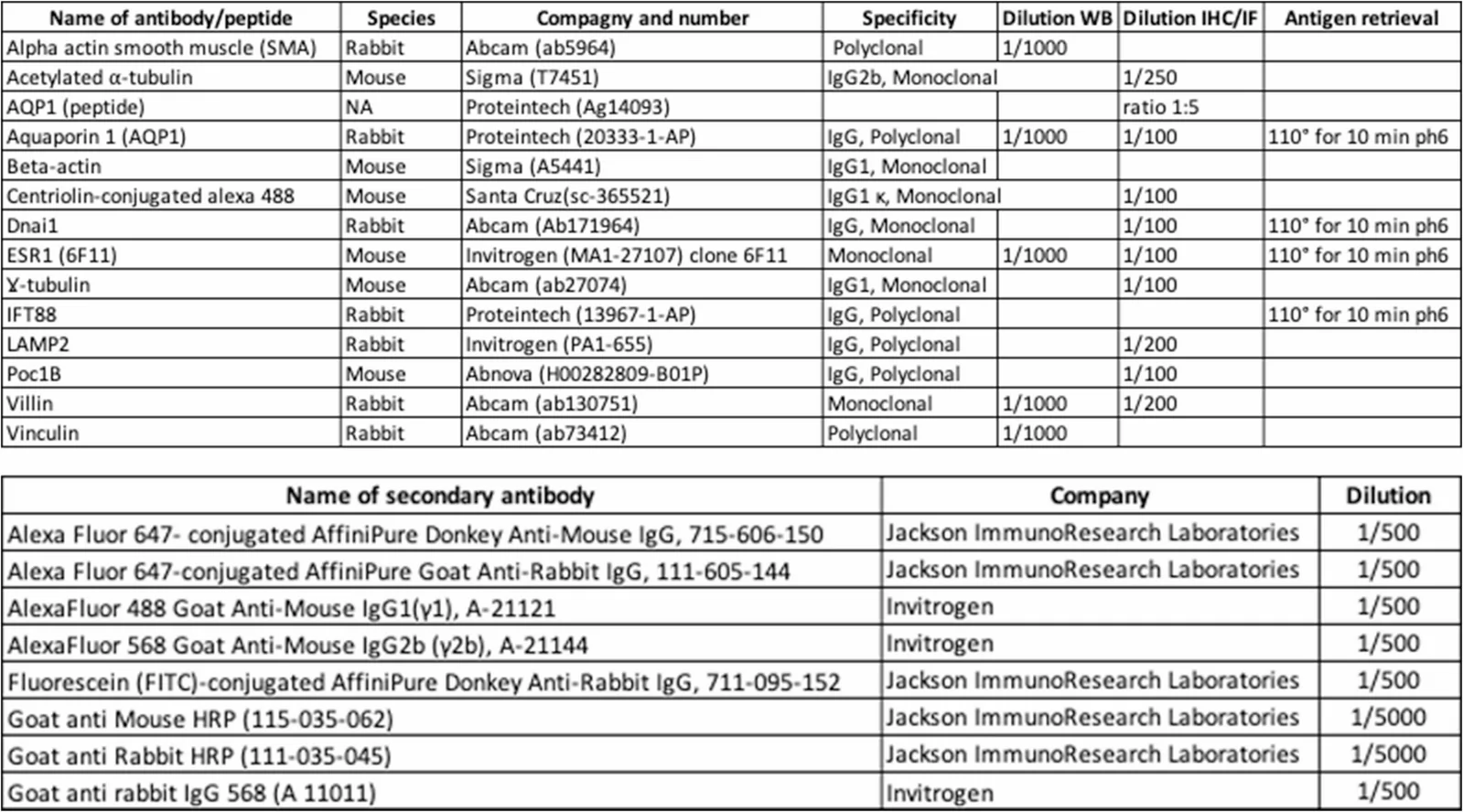
List of antibodies used in this study

## Cilia length measurement

Cilia length measurements were performed on motile cilia in efferent ductules. Samples were imaged using confocal microscopy and motile cilia were identified based on the detection of the dual markers acetylated tubulin and gamma tubulin and their characteristic organization into dense apical tufts. Z-stack images were acquired, and maximum intensity projections were generated. Cilia length was measured from the apical cell surface to the distal tip of the ciliary tuft using image analysis software ImageJ. Only clearly resolved tufts oriented in the imaging plane were included in the analysis. Multiple measurement for each tuft (minimum 3) and regions and biological replicates (*n* = 3) were analyzed to ensure reproducibility.

## Histologic evaluation

Hematoxylin and Eosin (H&E) staining was performed to assess the general morphology of the testis, efferent ductules, and epididymis. Paraffin-embedded tissue sections  (5–10 μm) were stained with Harris Modified Hematoxylin Solution (HHS32, Sigma-Aldrich) and counterstained with Eosin. Slides were imaged using a Zeiss Axioskop 2 Plus phase-contrast microscope (Zeiss, Oberkochen, Germany) equipped with a digital camera (Diagnostic Instruments, USA). Image acquisition was carried out using Spot software.

### Immunoblotting

Western blot analyses were performed using 20 µg of total protein extracted from the testis, total efferent ductules, proximal efferent ductules, and epididymis from Ctl and Ift88 cKO mice. Proteins were separated by SDS-PAGE and transferred onto PVDF or nitrocellulose membranes using a semi-dry transfer system for 30 min at 25 V (Trans-Blot Turbo, Bio-Rad). Membranes were blocked in 2.5% BSA for 1 h at room temperature, then incubated overnight at 4 °C with the primary antibody (Table 3). After three washes in PBS containing 0.05% Tween-20 (PBST), membranes were incubated with the appropriate HRP-conjugated secondary antibody for 1 h at room temperature. Following three additional washes in PBST, signal detection was carried out using a chemiluminescent substrate (Bio-Rad, #170–5061) and visualized with the ChemiDoc MP Imaging System (Bio-Rad). Band intensities were quantified using ImageLab software (version 6.0.1, Bio-Rad). Vinculin, smooth muscle actin (SMA) or β-actin were used as loading controls. Deglycosylation was carried out on total protein extracts from kidney and efferent ducts using PNGase F (P0704L, NEB) for 1 h at 37 °C. Samples were subsequently resolved by SDS-PAGE and immunoblotted with an anti-AQP1 antibody. Villin was used as a loading control.

### Transmission electron microscopy

Adult male reproductive systems of Ctl and Ift88 cKO mice were collected and fixed by immersion in 4% paraformaldehyde (PFA) immediately after sacrifice, for a maximum duration of 30 min. Efferent ductules were then carefully dissected and fixed overnight at 4 °C in a solution containing 0.05% malachite green, 4% paraformaldehyde, and 4% glutaraldehyde in 0.1 M sodium cacodylate buffer. Tissues were subsequently rinsed in 0.1 M sodium cacodylate buffer prior to further processing. Samples were then immersed for 2 h in a solution containing 1% osmium tetroxide and 1.5% potassium ferrocyanide. All tissues were dehydrated in a graded series of acetone and embedded in Epon 812 medium (Mecalab Ltd., Montreal, QC). Thin sections (100 nm) of embedded blocks of the efferent ductules were cut with a diamond knife and mounted on copper grids for EM analysis. They were stained for 5 min with uranyl acetate and 3 min with lead citrate and examined with a FEI Tecnai 12 120 kV Transmission Electron Microscope (Facility for Electron Microscopy Research, McGill University (QC), Canada), and a Tecnai G2 Spirit Transmission Electron Microscope (FEI Company) operated at 120 kV (Electron Microscopy Center 2.0, University of Siena, Italy).

### Computer assisted sperm analysis (CASA analysis)

Spermatozoa were allowed to swim out from cauda epididymidis of Ctl and Ift88 cKO adult mice after incubation in 500 µl of Whittens media (pH7.4 and osmolarity 300 mOsm) at 37 degrees, as previously described^7^. Total sperm count and sperm motility were analyzed following manufacturer instructions using the Microptic Nikon eclipse Ci (camera Basler acA1300-200uc (24031536)) operating with the software SCA V6.6.0.5 (Microptic, Barcelona, Spain).

### High speed videomicroscopy of cilia beating

Whole efferent ductules were freshly dissected to carefully remove the connective tissue under a microscope in Hanks’ Balanced Salt Solution (HBSS, SH30588; HyClone), as previously described^7^. Efferent ductules of adult mice were mounted on a culture dish with a round coverslip in a drop of HBBS (n°0420041500B, Bioptechs) and placed on an inverted microscope (Zeiss Axio Observer Z1) equipped with a warming plate set at 35 °C. Cilia beating was recorded with a high-speed camera (Hamamatsu ORCA-Flash4.0, n°C13440-20CU) at 63x magnification with oil immersion, capturing at a rate of 100 frames per second. At least six videos were analyzed for each sample.

### Statistical analysis

All experiments were conducted on 3 to 5 biological replicates per group (Ctl and Ift88 cKO mice). All replication attempts yielded consistent results. Data normality was assessed using the Shapiro-Wilk test and visual inspection of Q-Q plots. For comparisons between Ctl and Ift88 cKO groups, parametric data were analyzed using unpaired t-tests, while non-parametric data were evaluated using the Mann-Whitney U test. Statistical analyses were performed using GraphPad Prism software (version 10.1.1), with significance set at *p* < 0.05.

## Results

### The loss of IFT88 ciliary protein in efferent ductules results in shorter cilia

To investigate the role of cilia in the efferent ductules (Fig. 1A), we conditionally disrupted the *Ift88* gene in epithelial cells of these tubules under the control of the *Villin* promoter (Fig. 1B). The efficient depletion of IFT88 in both motile and primary cilia was confirmed by immunostaining and immunoblotting analyses. In control (Ctl) efferent ductules, IFT88 was detected along motile cilia in MCCs and primary cilia in RPCCs (Fig. 1C), whereas it was completely absent in efferent ductules from Ift88 cKO mice (Fig. 1C). Consistently, immunoblotting confirmed the loss of IFT88 protein in efferent ductule extracts from Ift88 cKO mice (Fig. 1D) in which motile cilia length was reduced by approximately 80% across all efferent ductule segments (proximal, conus, and distal) (Fig. 1E). In contrast, reliable assessment of primary cilia length was not feasible, as primary cilia were frequently obscured by the dense motile cilia staining. Despite the loss of IFT88, remnants of both motile and primary cilia were still detectable in Ift88 cKO efferent ductules (Fig. 1C, F). The presence of these residual cilia marked by acetylated α-tubulin staining above centriolar markers (Fig. 1C) and observed by Transmission Electron Microscopy (TEM) (Fig. 1F), suggests that the initial assembly of the ciliary base in both MCCs and RPCCs of the efferent ductules may occur independently of IFT88, as observed in other model systems [33]. Using the Cre-Lox system under the control of the Villin promoter, we effectively disrupted cilia formation by targeting IFT88 in cilia of the efferent ductules, without affecting IFT88 protein level in the neighboring reproductive tissues such as the testis and epididymis (Supplementary Fig. 1A, B).

### Disruption of IFT88 in cilia causes severe male reproductive defects and infertility

**Fig. 2:**
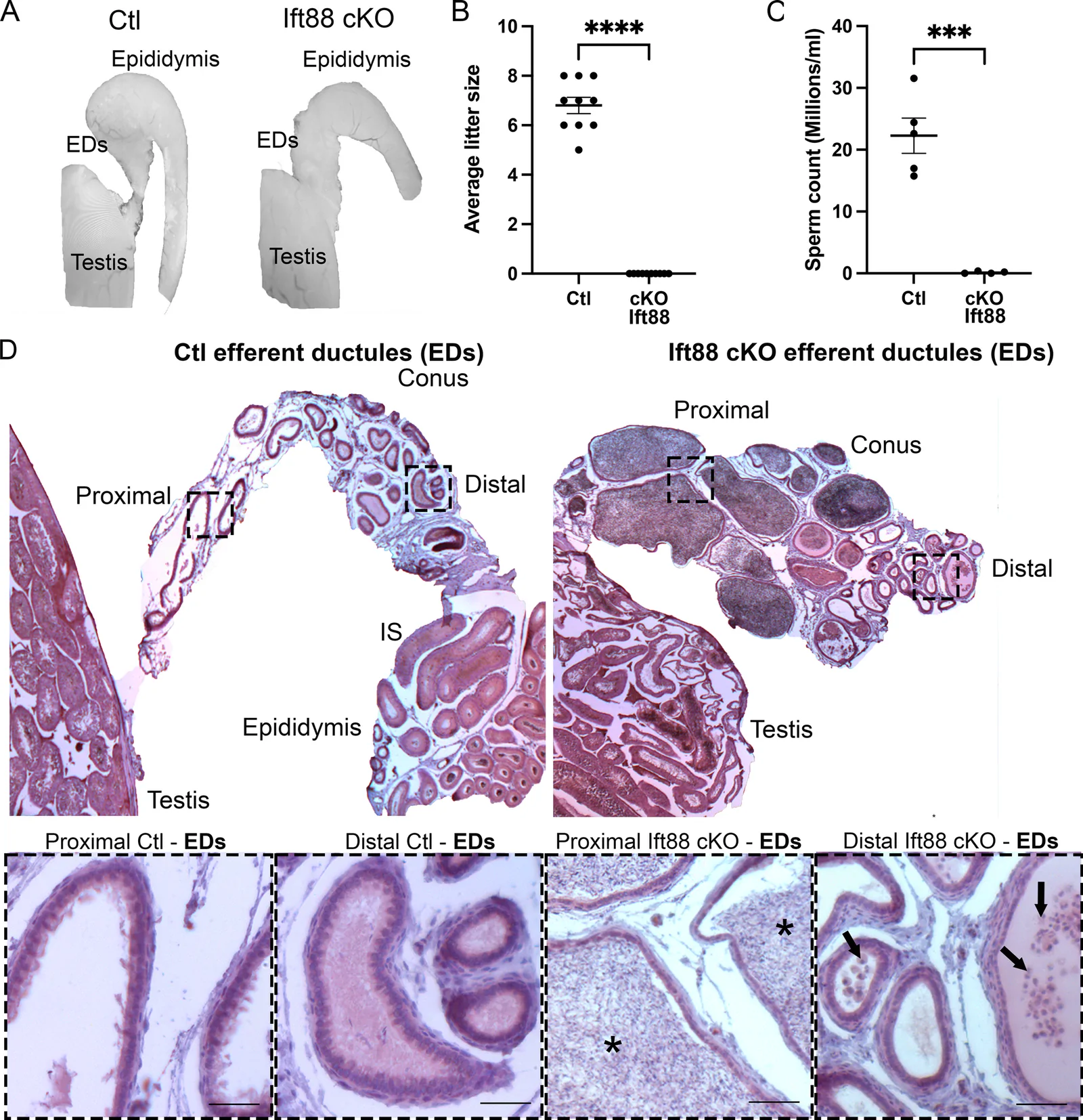
**Loss of IFT88 causes efferent ductule dilation and sperm accumulation leading to infertility**. **A **Macroscopic photos of male reproductive system in 10 weeks old Ctl and Ift88 cKO mice showing visible enlargement of efferent ductules. **B **Average litter size after mating adult Ctl or Ift88 cKO males (n = 5 per group, each mated with two WT females) is presented as mean ± standard deviation (SD). Mating was confirmed by detection of vaginal plugs in all females. **C **Sperm concentration (in Millions/ml) from cauda epididymides of adult Ctl (n=5) and Ift88 cKO (n=4) males was assessed using Computer-Assisted Sperm Analysis. **D **Hematoxylin and eosin staining of efferent ductules from 10-week-old mice shows proximal ductule enlargement and sperm accumulation in Ift88 cKO. Magnification of proximal and distal regions of the efferent ductules from both Ctl and Ift88 cKO mice. Asterisks indicate sperm accumulation in the proximal efferent ductule of Ift88 cKO. The conus and distal efferent ductule segments in Ift88 cKO lack sperm but contain ectopic round-shaped cells in the lumen (arrows). Scale bar: 20µm. *** *p*<0,001; **** *p *<0,0001. EDs: efferent ductules; IS: Initial segment

Both Ctl and Ift88 cKO male mice reached adulthood with no overt health defects, as expected. However, gross anatomical examination of the male reproductive system revealed a visible enlargement of the efferent ductules in adult Ift88 cKO males, detectable by the naked eye (Fig. 2A). Fertility testing showed that while Ctl males produced an average litter size of 6–7 pups when mated with WT females, Ift88 cKO males were infertile, failing to produce any offspring (Fig. 2B). To verify the cause of infertility, we analyzed cauda epididymal sperm counts using Computer-assisted semen analysis (CASA) and very few spermatozoa were detected in Ift88 cKO compared to Ctl (Fig. 2C, Supplementary Fig. 2). The few sperm detected were immotile and exhibited coiled flagella, likely caused by altered luminal fluid dynamics and impaired efferent ductule homeostasis [20].

Histological analysis of adult efferent ductules showed major morphological changes following IFT88 loss. Hematoxylin and eosin staining revealed enlarged tubules, particularly in the proximal efferent ductules, accompanied by epithelial thinning and sperm accumulation in this region (Fig. 2D). In contrast, the distal efferent ductules of Ift88 cKO male, adjacent to the epididymis, were devoid of sperm and showed ectopic round-shaped luminal cells in the lumen (Fig. 2D), consistent with luminal obstruction underlying obstructive azoospermia.

In adult Ift88 cKO mice, sperm accumulation in efferent ductules triggered backflow of fluid and sperm leading to rete testis enlargement compared to Ctl, ultimately altering the seminiferous tubules (Supplementary Fig. 3A, B). However, testis weights in Ift88 cKO males were not significantly different from those of Ctl mice at 10 weeks of age (Supplementary Fig. 3C). As expected, spermatozoa were present throughout all epididymal segments in adult Ctl mice, whereas epididymides from adult Ift88 cKO mice were mostly devoid of sperm (Supplementary Fig. 2A, B, D). In the sperm-depleted epididymides of Ift88 cKO mice, which were significantly lighter than those of controls, the corpus and cauda contained flocculent luminal secretions and a few non-spermatic cells (Supplementary Fig. 2B, C).

To determine whether sperm accumulation was responsible for the dilation of the efferent ductules and rete testis, we analyzed the morphology of these structures in prepubertal mice, before the initiation of sperm production. In Ctl mice, the rete testis and efferent ductules exhibited normal morphology. In contrast, Ift88 cKO mice showed enlargement of the rete testis and proximal efferent ductules, accompanied by the presence of round cells within the lumen (Supplementary Fig. 4). These findings indicate that enlargement of the efferent ductules and rete testis occurs independently of sperm production, as early as three weeks of age, suggesting an abnormal increase in fluid secretion into the lumen. In adult Ift88 cKO mice, the pronounced enlargement of the efferent ductules likely arises from defective trans-membrane fluid transport and sperm accumulation, resulting in elevated intraluminal pressure that may intensify with age. This increased pressure would contribute to backflow into the rete testis and structural disruption of the seminiferous epithelium in aged mice (Supplementary Fig. 5). Altogether, loss of IFT88 in the efferent ductules resulted in severe morphological abnormalities that disrupted transepithelial fluid transport from early developmental stages, ultimately compromising testicular integrity in the adult, due to the accumulation of fluid and sperm in the rete testis and seminiferous tubules of the testis. 

### *Ift88* deficiency impairs motile cilia function

To determine whether the infertility phenotype could result from intrinsic defects in sperm structure, we examined sperm flagella by transmission electron microscopy (TEM) (Fig. 2A). As expected, in both Ctl and Ift88 cKO mice, sperm flagella displayed a typical 9 + 2 axonemal organization, with no obvious structural abnormalities (Fig. 2A), consistent with the absence of Cre-lox recombination in the testis, which if present could have impaired flagellogenesis (Supplementary Fig. 6).These observations confirm that infertility could not be due to ultrastructural defects in the sperm flagellum after deletion of Ift88.

We therefore focused on efferent ductule motile cilia, whose beating is essential for luminal fluid dynamics, with impaired motility linked to luminal obstruction and sperm agglutination [1–4, 9]. Cilia motility depends on a conserved 9 + 2 axoneme, where dynein arms and DNAI1-containing complexes generate the force required for beating [39]. Effective function also requires proper basal body organization and basal foot-mediated planar orientation to ensure coordinated, directional flow [1, 40]. Since disruption of these structural and molecular elements leads to impaired ciliary motility we assessed these key determinants in our model.

We assessed motile cilia structural features and beating capacities in adult Ctl and Ift88 cKO mice using TEM, high-speed video and confocal microscopy (Fig. 2B-E). In Ctl mice, efferent ductules exhibited normal ciliary motility of MCCs, whereas no cilia movement was detected in Ift88 cKO efferent ductules (Fig. 2B and Supplementary videos 1, 2). DNAI1, a dynein axonemal intermediate chain required for the function of the outer dynein arms and ciliary motility [41–43] was used to evaluate ciliary structure. As expected, DNAI1 was localized along the length of motile cilia in Ctl efferent ductules but was absent in primary cilia of RPCCs. Notably, DNAI1 staining appeared more intense at the ciliary base and gradually weakened toward the tip (Fig. 2C). In Ift88 cKO efferent ductules, DNAI1 was still detected- though at reduced intensity- in some of the short cilia on MCCs, while remaining absent in primary cilia (Fig. 2C).

At the ultrastructural level, a typical 9 + 2 axonemal organization was observed in motile cilia from Ctl mice, including both outer and inner dynein arms and a central pair, all essential for ciliary beating (Fig. 2D). In contrast, the shortened Ift88 cKO cilia, whose transversal view was only rarely observed exhibited heterogeneous ultrastructural features. In some cross-sections, axonemal organization appeared altered, with irregular arrangements of microtubule doublets within the canonical 9-doublet ring (Fig. 2D). This suggests that axonemal organization may be altered in Ift88 cKO motile cilia, although the extent and nature of these defects are difficult to assess due to the pronounced cilia shortening in mutant samples, which limits the acquisition of well-defined TEM cross-sections.

To further characterize the defect in cilia beating, we analyzed the organization of the ciliary base using Poc1b and Centriolin as markers of basal bodies and basal feet, respectively [44] (Fig. 2E). By TEM and confocal microscopy we observed that in Ctl efferent ductules, basal bodies and basal feet were properly aligned, supporting coordinated and directional ciliary beating (Fig. 2D, E). In contrast, Ift88 cKO efferent ductules exhibited misoriented basal bodies and basal feet, indicating disrupted organization at the ciliary base (Fig. 2D, E).

Together, these results confirm the essential role of *Ift88* in both the formation and motility of efferent ductule motile cilia, whose loss, as in other ciliary models, leads to sperm agglutination within the lumen (Table 1).

### IFT88 is required for fluid exchange and AQP1 localization and glycosylation

Given the luminal enlargement in efferent ductules of the Ift88 cKO males, a common response in other models that targeted the function of RPCCs, we investigated whether IFT88 loss impairs fluid reabsorption in the efferent ductules.

**Fig. 3:**
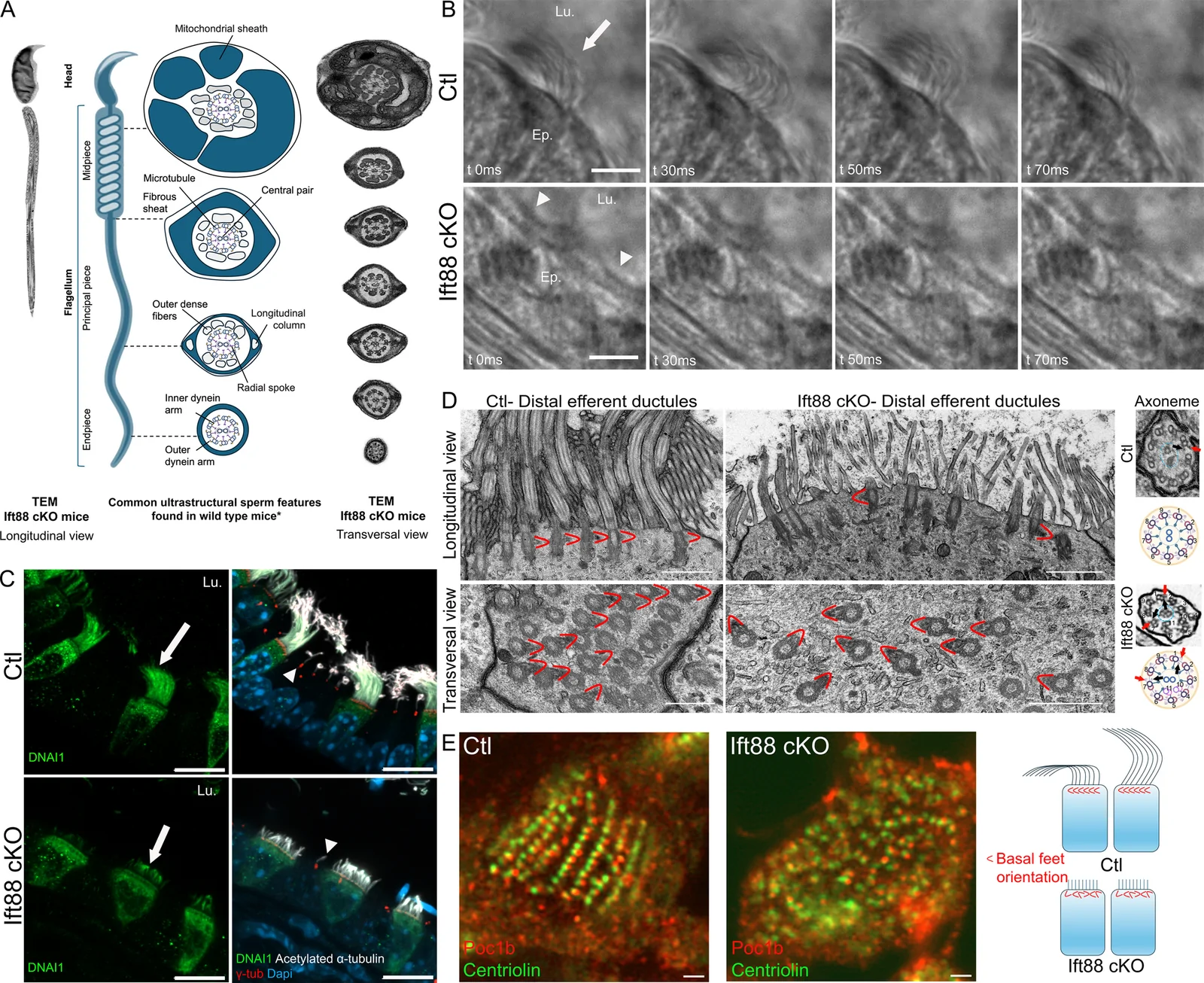
**Loss of IFT88 does not alter sperm flagellar ultrastructure but leads to non-functional residual motile cilia**. **A **Ultrastructure of spermatozoa agglutinated in the efferent ductules from Ift88 cKO mice was investigated by TEM and compared to the common ultrastructural features already described in spermatozoa from wild type mice. *Schematic view adapted from TEM observation made by Lindemann and Lesich, 2016 [82]. **B **Cilia beating was assessed by high-speed video microscopy (refer to associated supplementary videos 1 and 2). Arrows indicate motile cilia in Ctl efferent ductules at different time points, illustrating active movement. Arrowheads in Ift88 cKO mark residual, immotile cilia. Ep: Epithelium, Lu: Lumen. **C **Immunofluorescent detection of DNAI1 (green), a component of the outer dynein arm essential for motility, acetylated α-tubulin (white) marking cilia axoneme and Ɣ-tubulin (Ɣ-tub, red) marking centrioles/basal bodies in adult Ctl and Ift88 cKO efferent ductules. DNAI1 is detected in motile cilia (arrow) in Ctl mice but absent in Ift88 cKO. Primary cilia (arrowheads) are negative for DNAI1 in both genotypes. Scale bar: 10 μm. **D **Transmission electron microscopy (TEM) images of multiciliated cells showing basal foot orientation (red arrows) and axonemal cross-sections of motile cilia in Ctl and Ift88 cKO mice. Microtubule doublets are numbered 1–9 in Ctl and 1–11 in a rare instance of a motile cilia axonemal cross-section observed in Ift88 cKO mice. Red arrows indicate outer dynein arms present in both genotypes, while black arrows highlight inner dynein arms in Ctl and their absence in the axoneme of Ift88 cKO mice. Scale bar: 700 nm. E Airyscan immunofluorescence imaging of basal bodies (Poc1b, red) and basal feet (Centriolin, green) in Ctl and Ift88 cKO efferent ductules. In Ift88 cKO, both cilia directionality and motility features appear disrupted. Scale bars: 1 μm

To this end, we examined the fluid reabsorptive properties of the efferent ductule epithelium, focusing on key components involved in water transport. Transmission electron microscopy revealed an increased abundance of lysosomes in RPCCs from *Ift88* cKO mice compared to controls (Fig. 3A), a finding further supported by LAMP2 immunostaining and quantitative analysis by confocal microscopy (Fig. 3B, C). This indicates an increased need for cellular recycling to preserve efferent ductule homeostasis in the absence of IFT88. This likely reflects a compensatory response to defects in fluid reabsorption, as cells accumulate endocytic cargo and require enhanced degradation and recycling.

**Fig. 4:**
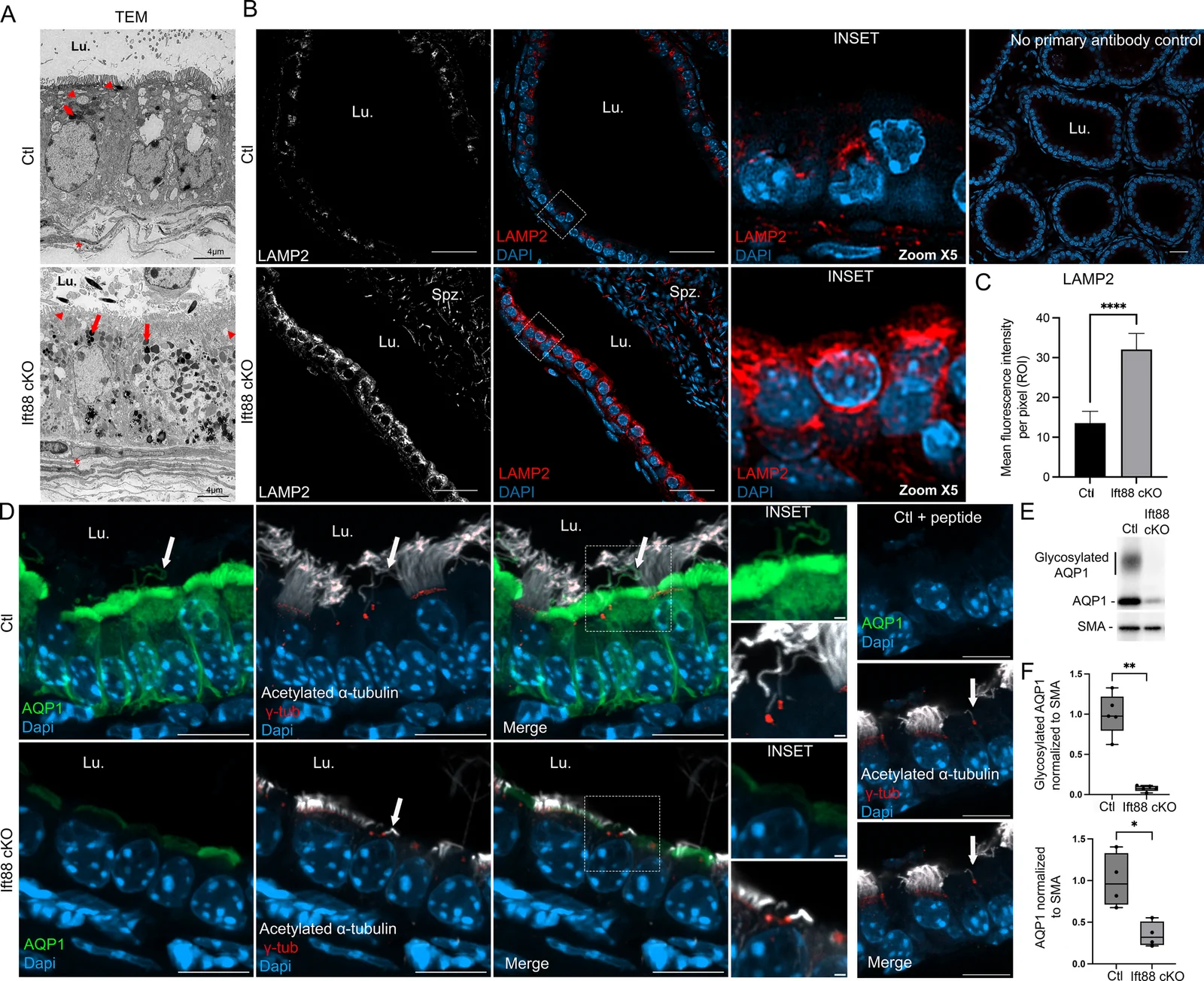
**IFT88 is required for AQP1 localization and glycosylation**. **A** Transmission electron micrograph (TEM) of efferent ductule epithelium in adult Ctl and Ift88 cKO mice. Red arrowheads indicate endocytic apparatus; stars highlight the underlying stroma composed of muscle cells and collagen. In reabsorptive primary ciliated cells from Ctl mice, occasional dense vesicles (putative lysosomes; red arrows) are visible, whereas Ift88 cKO cells show a noticeably higher abundance of these electron-dense vesicles (red arrows), suggesting increased lysosomal activity. Lu: lumen. **B** Representative confocal images of the conus segment of efferent ductules from control (Ctl) and Ift88 cKO mice stained with an antibody against LAMP2 to visualize lysosomes. In both genotypes, LAMP2-positive structures are predominantly distributed in the perinuclear region of epithelial cells. **C** Ift88 cKO mice exhibit a marked increase in LAMP2 fluorescence intensity compared to controls, indicating an expansion of the lysosomal compartment, which is consistent with ultrastructural findings obtained by transmission electron microscopy (TEM). **** *p *<0.0001, Mann Whitney test. Scale bars: 30 µm. **D** Immunofluorescent detection of AQP1 (green), a water channel highly expressed in the efferent ductules, acetylated α-tubulin (gray) marking cilia axoneme and Ɣ-tubulin (Ɣ-tub, red) marking centrioles/basal bodies in Ctl and Ift88 cKO mice. AQP1 is localized to the apical and basolateral membranes of primary ciliated cells and is also detected near the primary cilium itself (arrow, inset). **E** Peptide competition assay (1:10 ratio) confirms AQP1 staining specificity, with signal loss in apical, basolateral, and ciliary compartments (arrow). **F** Western blot analysis of AQP1 in ED protein extracts from Ctl and Ift88 cKO mice (n = 5 per group). Quantification of total and glycosylated AQP1 was normalized to Smooth Muscle Actin (SMA) and is shown in the right panels. * *p*<0.05, **<0.001, unpaired t-test. Scale bars: 10 µm

We next analyzed AQP1 distribution across different segments of the efferent ductules in Ctl and Ift88 cKO mice. AQP1 is one of the major player in water reabsorption found in RPCCs of the efferent ductules [45]. Consistent with previous studies, AQP1 was detected in the apical and basolateral membranes of RPCCs, as well as in endocytic vesicles [45, 46]. While apical localization was maintained in both genotypes, AQP1 staining in Ift88 cKO efferent ductules showed a diminished brush border signal and a complete loss of basolateral localization in the RPCCs (Fig. 4D). In addition, our data show that AQP1 is specifically localized to RPCCs and is detected in close proximity to the primary cilium itself (Fig. 4D, Supplementary Videos 3, 4, 5, 6), as validated by the loss of staining following AQP1 antibody preimmune competition (Fig. 4D, right panels). Overall, Ift88 cKO mice show reduced apical AQP1 levels in RPCCs and no detectable localization to primary cilia.

Immunoblotting revealed two different molecular weights for AQP1, suggesting posttranslational modifications (Fig. 4E). Since AQP1 is known to be glycosylated in the rat kidney, we performed enzymatic deglycosylation on efferent ductules protein extracts to assess its modification state (Supplementary Fig. 7). In Ctl samples, AQP1 appeared as a sharp band at 25 kDa and a diffuse band between 35 and 40 kDa. Following deglycosylation, the higher molecular weight form disappeared, confirming it as the glycosylated isoform (Supplementary Fig. 7). In the efferent ductules of Ift88 cKO male we identified a significant reduction of AQP1 and an almost complete absence of the glycosylated form (Fig. 4F), suggesting that glycosylated AQP1 is intimately related to the basolateral and primary cilia location of this water channel.

Together, these results demonstrate that IFT88 is required for proper AQP1 localization and that AQP1 glycosylation is directly or indirectly dependent of IFT88. Because AQP1 expression is also diminished in the efferent ductules of estrogen receptor 1 (ESR1) knockout mice [47], we next examined whether cilia loss in Ift88 cKO males could influence ESR1 expression or localization to better understand the mechanistic reduction of AQP1 staining. 

### Loss of IFT88 leads to progressive disorganization of proximal efferent ductules via disruption of estrogen signaling and microvilli structure

**Fig. 5:**
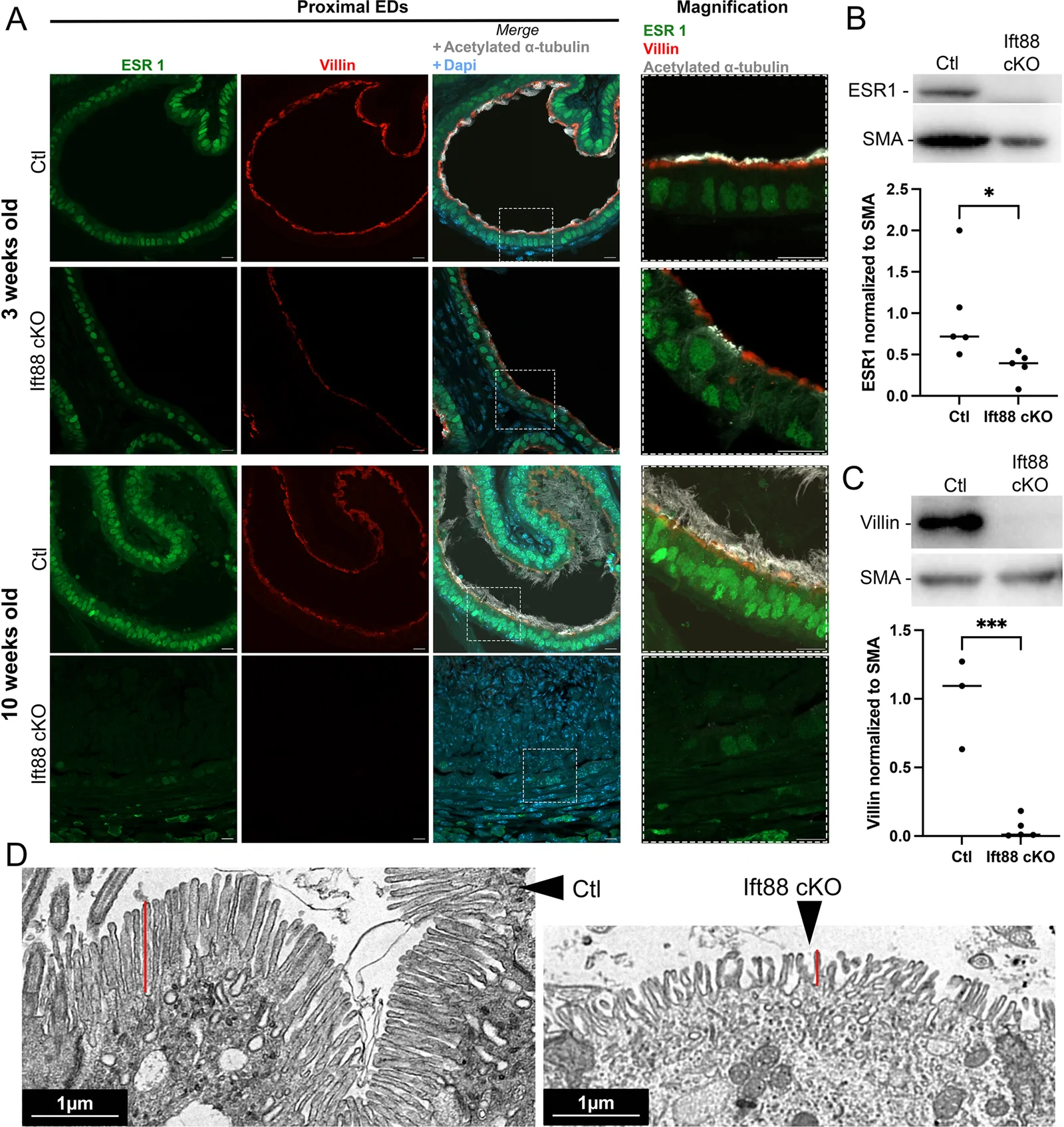
**Disruption of IFT88 compromises epithelial features essential for fluid reabsorption**. **A** Immunofluorescent staining of Villin (red, an actin-associated microvillar protein), estrogen receptor alpha (ESR1, green), and cilia axoneme (acetylated α-tubulin, white) in the proximal efferent ductules of Ctl and Ift88 cKO mice at 3 and 10 weeks of age. At 3 weeks, nuclear ESR1 is present in epithelial cells of both genotypes. By 10 weeks, ESR1 signal is markedly reduced in the proximal efferent ductules of Ift88 cKO mice. Villin shows clear apical localization in both groups at 3 weeks but is retained only in Ctl mice at 10 weeks. Scale bars: 10 µm. **B** Western blot analysis of ESR1 expression in efferent ductule protein extracts from Ctl and Ift88 cKO 10 weeks old mice and quantified after normalization to SMA (n = 3 Ctl; n = 5 Ift88 cKO; center line represents median). * *p *<0.05, Mann-Whitney test. **C** Western blot detection of Villin in proximal ED extracts from Ctl and Ift88 cKO 10 weeks old mice, normalized to SMA (n = 5 per genotype; center line: median). ***<0.0001, unpaired t-test. **D** Transmission electron microscopy showing microvilli length (highlighted by red lines) in proximal ED RPCCs from Ctl and Ift88 cKO mice

Estrogen receptor signaling is known to support the physiological function of the efferent ductules, particularly through its role in fluid reabsorption [5, 28, 47, 48]. To explore a potential link between cilia function and estrogen receptor pathways, we examined the localization of ESR1 in the efferent ductules from Ctl and Ift88 cKO mice (Fig. 5A-C, Supplementary Fig. 8). In Ctl mice, ESR1 was localized in nuclei of immature epithelial cells of the efferent ductules as early as 3 weeks of age and remained present at 10 weeks old throughout all segments of the efferent ductules (Fig. 5A, Supplementary Fig. 8). After the loss of IFT88, no major differences were observed in prepuberal mice. However, in adults, we identified a significant reduction of ESR1 detection in the proximal efferent ductule region, which was further confirmed by western blot analysis of ESR1 in these ductules from Ctl and Ift88 cKO mice (Fig. 5A, B). In contrast, ESR1 remained detectable in the conus and distal efferent ductules of Ift88 cKO mice, indicating that IFT88 loss impacts the efferent ductule segments differentially (Supplementary Fig. 8). Although ESR1 is initially expressed in prepuberal Ift88 cKO mice, the loss of cilia appears to result in reduced or absent nuclear ESR1 localization in adult Ift88 cKO mice, specifically in the proximal efferent ductules. This progressive loss of ESR1 may contribute to disrupted estrogen-mediated fluid reabsorption pathways in adult Ift88 cKO mice.

To further investigate the efferent duct enlargement phenotype observed in Ift88 cKO mice as early as 3 weeks of age, we examined the localization of Villin, an actin-binding protein involved in microvilli structure and specifically present in RPCCs of the efferent ductules^7^. In Ctl mice, Villin was consistently detected at the apical membrane of RPCCs in all three efferent ductule segments, both in prepuberal and adult animals (Fig. 5A, Supplementary Fig. 8). Similarly, Villin localization was preserved in all efferent ductule segments of 3-week-old Ift88 cKO mice. However, in adult Ift88 cKO mice, Villin protein was no longer detectable by immunofluorescence and western blot (Fig. 5A, C, Supplementary Fig. 8). Transmission electron microscopy of proximal efferent ductules further revealed a reduction in microvilli length in Ift88 cKO mice compared to controls (Fig. 5D).

Taken together, these findings indicate that the loss of ESR1 and Villin, along with shortened microvilli-particularly in the proximal efferent ductules of adult Ift88 cKO mice-reflects a progressive deterioration of epithelial architecture.

## Discussion

In the male reproductive tract, efferent ductules are specialized tubules that facilitate fluid reabsorption and transport sperm from the testis to the epididymis. They feature an epithelial lining composed of two principal cell types: multiciliated cells (MCCs) and reabsorptive primary ciliated cells (RPCCs) [7, 11, 16]. MCCs contain numerous motile cilia, which have been recognized for their role in generating luminal turbulence, which is critical for preventing sperm aggregation and maintaining fertility^1^. In contrast, the functions of non-motile primary cilia on RPCCs have received limited attention, even though primary cilia are well-known to regulate cellular homeostasis and signaling pathways in diverse organ systems [13, 49]. Through targeted disruption of *Ift88* - a gene coding for a protein indispensable for the assembly of both motile and primary cilia - in the efferent ductule epithelium, our findings highlight the synergistic roles of motile cilia and primary cilia in supporting ductal physiology, epithelial integrity, as well as fertility. By juxtaposing the phenotype of the Ift88 conditional knockout model with those exhibiting selective impairment of motile cilia or RPCC functions (Table 1), we have gained initial insights into the distinct contributions of each ciliary type within the MCCs and RPCCs in the efferent ductules homeostasis and male fertility.

### IFT88 controls both assembly and structural integrity of efferent ductule cilia

IFT88 is essential for ciliary assembly and structural integrity in efferent ductules. Initially identified in Chlamydomonas and mice, IFT88 deletion impaired flagella and cilia formation, resulting in shortened kidney cilia in mice [33]. As a core component of the anterograde IFT-B complex, IFT88 is essential for ciliary assembly across eukaryotes [33, 50–52]. IFT88 knockout models are widely used to study ciliary function because, in these models [33, 51, 52], cilia fail to extend beyond the transition zone, a structure that normally lies above the basal body and spans only 0.15-1.5 µm [53]. Consistent with previous studies [51, 52], conditional IFT88 knockout (cKO) in the efferent ductules dramatically shortened both motile and primary cilia. However, unlike those studies, the residual ciliary stubs observed in our model retained acetylated α-tubulin immunoreactivity - a hallmark of stable microtubules - suggesting partial preservation of the axoneme. Notably, these residual ciliary stubs, measuring approximately 3 μm, suggest that limited axonemal extension can occur beyond the transition zone. Motile cilia exhibited no beating after the loss of IFT88. DNAI1, an outer dynein arm (ODA) protein remained detectable in the shortened motile cilia, matching TEM observations of intact ODAs. This suggests that DNAI1 localization as well as ODA assembly and maintenance in efferent ductule motile cilia is IFT88-independent. However, inner dynein arms (IDAs) were unevenly distributed, being absent from some microtubule doublets while retained in others. Although IFT88 has not previously been linked to inner dynein arm defects, these observations suggest a potential role in their proper assembly or organization. One possible explanation is differential dependency on IFT cargo adapters: ODAs and IDAs use distinct adapters (ODA16 and IDA3, respectively), with ODA16 potentially retaining partial interaction with IFT trains even in the absence of IFT88, whereas IDA3 may require an intact IFT-B complex for efficient cargo binding and delivery. This could account for the heterogeneous impact on inner dynein arm organization, highlighting a context-dependent and incompletely understood contribution of IFT88 to motile cilia ultrastructure in efferent ductules. Consistently, some IFT88 cKO motile cilia also displayed aberrant axonemal organization, including 11 + 2 microtubule arrangements, suggesting a broader role for IFT88 in ciliary elongation and in maintaining the canonical 9 + 2 structure. Comparable defects, including extra microtubule doublets, occur in *Ift88* knockout sperm flagella [54]. Similar to ependymal motile cilia [55], where IFT88 ensures basal body and basal foot alignment, the IFT88 cKO showed misoriented basal feet, possibly from cytoskeletal disruptions or absent mechanical/fluid-flow signals within MCCs [56]. Overall, our findings indicate that IFT88 not only plays a key role in ciliogenesis but also contributes to the assembly and structural organization of both motile and primary cilia in the efferent ductules, impacting axonemal integrity, inner dynein arm distribution, and basal body orientation at this site.

### Loss of motile cilia causes luminal obstruction and reveals early RPCC dysfunction

To better understand the consequences of impaired motile cilia growth and function in Ift88 cKO males, comparisons with other animal models were made to identify pathologies specifically associated with MCC defects (Table 1). Ift88 cKO males exhibited obstructive azoospermia, similar to phenotypes observed in motile cilia-specific models [1–4], resulting from sperm agglutination within the efferent ductule lumen. These occlusive masses blocked sperm transit to the initial segment of the epididymis, leading to infertility characterized by the absence of sperm in the cauda epididymis and secondary fluid accumulation in the efferent ductules, rete testis, and seminiferous tubules.

Luminal obstruction is a hallmark of motile cilia deficiency in males and differs markedly from defects caused by RPCC dysfunction, such as in ESR1 KO mice, where all efferent ductules regions - from the rete testis to the distal efferent ductules segment - are dilated but remain patent [48, 57]. In MCC-targeted models, the lack of luminal mixing is thought to impair sperm-surface interactions and hinder epithelial endocytosis of Sertoli cell-derived proteins such as Clusterin, which normally bind sperm membranes upon testicular exit and are subsequently removed by epithelial uptake [37]. Loss of motile cilia thus prevents fluid agitation, leading to stagnation, sperm aggregation, and eventual occlusion, consistent with reports that motile cilia generate turbulence within the efferent ductule lumen [1–4].

However, the timing of rete testis dilation in Ift88 cKO mice indicates that MCC loss is not the sole contributor to the phenotype. When only motile cilia are disrupted, as in Foxj1-cre or DNAH5 KO models [1–4], rete testis dilation occurs only after substantial sperm accumulation and luminal blockage in the efferent ductules, coinciding with the first wave of sperm release around postnatal day 30–35 (Table 1). This supports the notion that rete testis dilation in these models results from sperm obstruction and backflow rather than impaired fluid reabsorption. By contrast, in Ift88 cKO mice, where both motile and primary cilia are absent, rete testis dilation appeared much earlier by postnatal day 21, suggesting a primary contribution from RPCC dysfunction to early fluid homeostasis defects.

This is further supported by observations from mouse models targeting genes essential for MCC function that are also expressed in RPCCs. For example, deletion of the cell cycle regulators E2F4/5 eliminates MCCs in the respiratory tract and efferent ductules but also affects RPCC function [ 37, 58], as evidenced by reduced ESR1, AQP1, and microvilli - features reminiscent of the ESR1 KO and overlapping with the Ift88 cKO phenotype. In both E2F4/5 KO and Ift88 cKO models, luminal occlusions arise after the first spermatogenic wave due to loss of motile cilia, whereas early-onset rete testis dilation occurs at neonatal to prepubertal stages, preceding tubule occlusions and implicating RPCC defects as an additional contributor to the observed male infertility phenotype.

### Impairment of the primary cilium leads to early luminal fluid accumulation

RPCCs, characterized by long apical primary cilia [7, 11], express key ion channels and transporters, including CFTR, AQP1, NHE3 (SLC9A3), AE2 (SLC4A2) [10, 45, 46, 59, 60], which are crucial for maintaining luminal fluid homeostasis and ductule morphology under the control of estrogen signaling [10, 48]. Through this fluid transport activity, they enable an approximately 28-fold increase in sperm concentration, ensuring efficient transport to the epididymis. Conditional deletion of IFT88 in efferent ductules epithelial cells impaired primary cilium growth and led to hallmarks of fluid transport defects, including tubular dilation, reduced epithelial height, alterations in microvilli length, and changes in AQP1, Villin and ESR1 protein expression. These defects were both region- and age-dependent, with reabsorptive impairments most pronounced in the proximal segment and progressively worsening from an early prepubertal age, before sperm release, to adulthood when sperm become agglutinated and block the ductules.

The proximal versus distal differences in reabsorptive phenotypes -reflected by variations in microvilli length, Villin and ESR1 protein expression -may partly stem from differences in mesonephric tubule origin and from the higher RPCC-to-MCC ratio characteristic of proximal efferent ductules, which are situated near the rete testis and specialized for fluid reabsorption [6, 61]. The timing of the emergence of this impaired reabsorptive phenotype coincides with the postnatal functional transition of the efferent ductules, which shift from an early secretory phase (P16 to puberty)-characterized by the production of signaling molecules, growth factors, and osmolytes that support epithelial differentiation and morphogenesis- to a predominantly reabsorptive role at puberty and thereafter, essential for sperm concentration and transport to the epididymis [62, 63].

At the prepubertal stage, the efferent ductules and rete testis of Ift88 cKO males were already markedly dilated, indicating that this early phenotype results from a primary effect on fluid transport, which is secretory before puberty, rather than from secondary effects such as sperm agglutination or tubular obstruction as observed in MCC-targeted models [1–4]. This observation likely reflects impaired signaling due to the absence of functional primary cilia on RPCCs. In addition, the calcium channels Polycystin-1 and - 2 (PKD1 and PKD2) are expressed in the epithelial cells of Wolffian duct and mesonephric tubules, the precursors of the efferent ductules, as early as E11.5–E12.5, although their specific association with RPCCs has not been clearly established [64–66]. Supporting the involvement of primary cilia in regulating fluid homeostasis, deletion of Pkd1 and Pkd2, key regulators of calcium channel activity and mechanosensation in renal primary cilia [67–69] leads to massive dilation of mesonephric tubules, as early as E13.5–15^65,66^. Their ablation in fetal or neonatal mice similarly causes abnormal epithelial responses, including excessive fluid secretion into the lumen during the developmental stage when the efferent ductule epithelium remains secretory rather than reabsorptive. Consistently, Pkd1⁻/⁻;Pax2-Cre mice display luminal dilation of the efferent ductules at P1, and Pkd1/2 knockouts exhibit epithelial flattening [65, 66] - a hallmark of reduced RPCC function- also observed in Ift88 cKO and Esr1-deficient models [28–32]. Supporting this, LGR4 (Leucine-rich repeat-containing G-protein coupled receptor 4) is a receptor that regulates oviductal epithelial secretion through the WNT signaling pathway [70], a pathway that has also been linked to primary cilia in other models [13]. LGR4 KO mice exhibit efferent ductule dilation in adulthood associated with reduced epithelial height, and the rete testis is already dilated by puberty [71]. Notably, such dilation and epithelial flattening is absent in models where only motile cilia are disrupted, underscoring the most probable role of primary cilia in controlling the secretory and reabsorptive functions of RPCCs respectively during the early postnatal and adult ages.

Although primary cilia have not been selectively deleted in adult efferent ductules, the present Ift88 cKO model, which eliminates both motile and primary cilia, provides a means to begin distinguishing their respective roles. Altogether, by comparing the Ift88 cKO model with those affecting only motile cilia or broadly impairing RPCCs, such as ESR1 KO mice [28–32], our work supports the hypothesis that motile cilia are primarily responsible for preventing sperm agglutination and obstruction at puberty, whereas primary cilia, as key signaling organelles, support the maintenance of luminal fluid balance during development and throughout adulthood. However, although no definitive conclusions can be drawn, our findings suggest that loss of Ift88 may disrupt cilia-dependent signaling in RPCCs (possibly through cAMP/CFTR-dependent transport), contributing to altered luminal fluid handling and a broader breakdown of epithelial organization and homeostasis, rather than a pathway-specific or transcriptionally driven effect.

### Mechanisms linking primary cilia, estrogen signaling, and AQP1

In adult mice, loss of IFT88 in the present study led to cystic pathology, characterized by luminal dilation, reduced epithelial height, diminished microvilli, and shortened cilia -phenotypes regulated by ESR1 and consistent with impaired fluid reabsorption observed in other models (Table 1). Loss of membrane ESR1 [72] phenocopies ESR1 KO [28–32] and RPCC/primary cilia models (Table 1), linking ESR1-mediated fluid reabsorption to primary cilia in adult efferent ductules through molecular mechanisms that await elucidation. AQP1, an ESR1-dependent water channel in the male reproductive tract [46], mediates trans-membrane water flux, a conserved activity across tissues and species [45, 73–76]. However, folding, stabilization and trafficking of some aquaporins [77], including AQP1 [78], is regulated by N-glycosylation [77, 79, 80]; therefore, we determined both localization and post-translational status of AQP1. We confirmed AQP1 localization at the apical and basolateral membranes of RPCC in Ctl mice, consistent with previous reports^45^,^46^, and further suggests its novel presence in close proximity with the primary cilia compartment in efferent ductules. While the protein appeared reduced at the brush border, no signal was detected at the basolateral membranes, and no clear association with the remnant primary cilium of RPCCs could be resolved in Ift88 cKO efferent ductules under our imaging conditions. In addition, we showed near-total depletion of the glycosylated AQP1, suggesting direct or indirect contribution of IFT88 in AQP1 posttranslational modification and its subcellular distribution, including potential association with the primary cilium. The proximity of AQP1 signal to the primary cilia compartment is reminiscent of observations in the nucleus pulposus, where AQP4 was identified in primary cilia of epithelial cells and implicated in rapid osmotic adaptation that involves the coordinated action of AQP1/AQP4 and the mechanosensitive cation channel TRPV4 (Transient receptor potential vanilloid 4) for water permeability, Ca²⁺ influx, and volume changes [81]. AQP1’s potential function in cilia requires further scrutiny, as a similar network may regulate osmoregulation/reabsorption in efferent ductules. The precise subcellular distribution and functional role of AQP1 within ED primary cilia also remains to be determined, including whether it can transport water across the ciliary membrane, or manage the molecular flux within such a confined compartment, and how these molecules are subsequently processed.

## Conclusion

Our study demonstrates that conditional deletion of *Ift88* in efferent ductules disrupts both motile and primary cilia of MCCs and RPCCs, respectively, profoundly affecting ductal physiology and male fertility. We also demonstrate that motile cilia prevent sperm obstruction, while primary cilia are essential for the postnatal switch from secretion to reabsorption, coordinating epithelial organization, aquaporin trafficking, and estrogen signaling in an age- and ductal location-dependent manner. These findings establish ciliary coordination as a critical determinant of efferent ductule homeostasis, reproductive health and function, with broader implications for understanding human infertility and ciliopathies arising from disrupted ciliary signaling and fluid regulation.

## Supplementary Information

Supplementary Material 1.

Supplementary Material 2.

Supplementary Material 3.

Supplementary Material 4.

Supplementary Material 5.

Supplementary Material 6.

Supplementary Material 7.

## Data Availability

The datasets generated during and/or analyzed during the current study are available from the corresponding author on reasonable request.

## Abbreviations

Ift88 cKO: (Ift88 conditional KnockOut)
Ctl: Control
MCC: Multi-Ciliated cells
RPCC: Reabsorptive Primary Ciliated cells
