# Supplementary material for: Primary and motile cilia in the efferent ductules: a role for IFT88 in maintaining male fertility
Source: Cell Mol Life Sci. 2026 Jun 23;83(1):323. doi: 10.1007/s00018-026-06296-w (PMC13547203; doi:10.1007/s00018-026-06296-w)
Supplement: Supplementary file 1 [file 18_2026_6296_MOESM1_ESM.docx]

**Supplementary Figures:**

**Supplementary Fig.1 Loss of IFT88 in Villin-Cre mice does not impair IFT88 detection in the testis and the epididymis. (A-B)** Western blot detection of IFT88 in protein extracts from testis **(A)** and epididymis **(B)** of Ctl and Ift88 cKO mice. β-actin (testis) and Smooth Muscle Actin, SMA (epididymis) were used as loading controls (left panels). Quantification of IFT88 levels was performed on samples from n = 5 mice per genotype and normalized to the respective loading control (right panels; center line: median). Statistical analysis was done using the Mann-Whitney test for panel A and unpaired t-test for panel B. ns: not significant.

**Supplementary Fig.2 The epididymis is mostly devoid of spermatozoa following the loss of IFT88 in the efferent ductules. (A)** Hematoxylin and eosin staining of epididymides from Ctl and Ift88 cKO at 10 weeks old. EDs: Efferent ductules; VD: Vas Deferens. **(B)** In control mice, the caput, corpus, cauda, and vas deferens are filled with spermatozoa (indicated by stars). In contrast, sperm are absent in all segments of the epididymis in Ift88 cKO mice and the lumen contains flocculent luminal secretion. Scale bars: 50µm **(C)** Epididymis weight, normalized to body weight, shows a significant reduction in Ift88 cKO mice compared to controls (n = 3 for Ctl, n = 5 for Ift88 cKO). **** p<0,0001. **(D)** CASA analysis of sperm isolated from the epididymis of Ctl and Ift88 cKO mice. Trajectories of motile sperm are shown in red and immotile sperm are highlighted in yellow. In the right panel, two immotile spermatozoa from Ift88 cKO mice are highlighted (arrow).

**Supplementary Fig.3 The loss of IFT88 in efferent ductules triggers rete testis enlargement and sperm backflow associated with seminiferous tubules defect. (A)** Hematoxylin eosin staining of testis from Ctl and Ift88 cKO at 10 weeks old. Ift88 cKO rete testis is enlarged with an accumulation of sperm cells (star). EDs: Efferent ductules. **(B)** Magnification of seminiferous tubules showed alteration of spermatogenesis in some seminiferous tubules in Ift88 cKO (stars). **(C)** Testis weight is not significantly different between Ctl and Ift88 cKO. ns: non-significant.

**Supplementary Fig.4 The loss of Ift88 trigger rete testis and efferent ductules enlargement in prepuberal mice. (A)** Hematoxylin and eosin staining of rete testis (RT) from Ctl and Ift88 cKO at 3 weeks old. Ift88 cKO rete testis is enlarged with an accumulation of ectopic round-shaped luminal cells (arrow). **(B)** Hematoxylin and eosin staining of efferent ductules from Ctl and Ift88 cKO at 3 weeks old. Higher magnification of the blue dotted area highlights the proximal tubules in both Ctl and Ift88 cKO samples. Arrow in Ift88 cKO indicates tubule enlargement in Ift88 cKO accompanied by the presence of round cells within the lumen. ST : Seminiferous tubules. Scale bars : 50µm.

**Supplementary Fig.5 Pronounced luminal backflow and absence of sperm in the epididymis of aged Ift88 cKO mice. (A)** Hematoxylin and eosin staining of testes from Ctl and Ift88 cKO at 10 months old. Arrows indicate the rete testis (RT) in both groups. In Ift88 cKO mice, the rete testis is notably enlarged and filled with accumulated spermatozoa. **(B)** Hematoxylin and eosin staining of epididymis from Ctl and Ift88 cKO at 10 months old with magnified views of the caput, corpus, and cauda regions. Spermatozoa are only visible in all segments of the Ctl epididymis (stars) and not in Ift88 cKO mice. Scale bars: 50µm.

**Supplementary Fig.6 In situ detection of recombination activity in the testis and efferent ductules under the Villin-Cre driver.** Immunofluorescent detection of endogenous red fluorescence (RosaTDtomato) and nuclei (DAPI) in testis and EDs from Vil1^Cre^; Ai14 strain. A strong reporter signal is observed in the EDs, whereas no detectable fluorescence is present in the testis.

**Supplementary Fig.7 Glycosylation status of AQP1 revealed by enzymatic assay.** AQP1 protein detection following deglycosylation of total protein extracts from efferent ductules using PNGaseF (lanes 1–3). In the absence of the enzyme (lanes 2–3), both glycosylated and non-glycosylated forms of AQP1 are detected, whereas the diffuse glycosylated band is absent in the presence of active PNGaseF (lane 1). Kidney samples (lanes 4–6) were included as positive controls for the deglycosylation assay, confirming the loss of the glycosylated AQP1 form upon incubation with PNGaseF at 37°C. Villin was used as a loading control.

**Supplementary Fig.8 Loss of apical Villin localization in distal efferent ductules of Ift88 cKO mice with age.** Immunofluorescent staining of Villin (red, an actin-associated microvillar protein), estrogen receptor alpha (ESR1, green), and cilia (acetylated α-tubulin, white) in the conus and distal efferent ductules (closer to the epididymis) of Ctl and Ift88 cKO mice at 3 and 10 weeks of age. At 3 and 10 weeks, nuclear ESR1 is present in epithelial cells of both genotypes. Villin shows apical localization in both groups at 3 weeks but is retained only in Ctl mice at 10 weeks in conus and distal segments. Scale bars: 10 µm.

**Supplementary video 1 Highspeed videomicroscopy of multiciliated cells from Ctl adult mouse efferent ductules.** Live recordings of efferent ductules from Ctl mice captured at a rate of 100 frames per second were converted to slow down playback to 15 frames per second at a magnification of 63x.

**Supplementary video 2 Highspeed videomicroscopy of multiciliated cells from Ift88 cKO adult mouse efferent ductules.** Live recordings of efferent ductules from Ift88 cKO mice captured at a rate of 100 frames per second were converted to slow down playback to 15 frames per second at a magnification of 63x.

**Supplementary video 3 Water channel AQP1 is located within primary cilium compartment in Ctl efferent ductules.** Three-dimensional confocal video reconstruction of Ctl efferent ductules showing the localization of AQP1 within the primary cilium compartment. Immunofluorescence staining with acetylated α-tubulin (magenta) marks ciliary axonemes, γ-tubulin (yellow) marks basal bodies, AQP1 (cyan), and nuclei are labeled with DAPI (white). Images were acquired at 63× magnification with a 4× digital zoom.

**Supplementary video 4 AQP1 localizes to the primary cilium in Ctl efferent ductules.** Three-dimensional confocal video reconstruction of Ctl efferent ductules showing the localization of AQP1 within the primary cilium compartment. Immunofluorescence staining with acetylated α-tubulin (magenta) marks ciliary axonemes, γ-tubulin (yellow) marks basal bodies, AQP1 (cyan), and nuclei are labeled with DAPI (white). Images were acquired at 63× magnification.

**Supplementary video 5 Loss of AQP1 from the remnant primary cilium in Ift88 cKO efferent ductules.** Three-dimensional confocal video reconstruction of Ift88 cKO efferent ductules showing the absence of AQP1 within the primary cilium compartment. Immunofluorescence staining with acetylated α-tubulin (magenta) marks ciliary axonemes, γ-tubulin (yellow) marks basal bodies, AQP1 (cyan), and nuclei are labeled with DAPI (white). Images were acquired at 63× magnification.

**Supplementary video 6 Loss of AQP1 from the remnant primary cilium in Ift88 cKO efferent ductules** Three-dimensional confocal video reconstruction of Ift88 cKO efferent ductules showing the absence of AQP1 within the primary cilium compartment. Immunofluorescence staining with acetylated α-tubulin (magenta) marks ciliary axonemes, γ-tubulin (yellow) marks basal bodies, AQP1 (cyan), and nuclei are labeled with DAPI (white). Images were acquired at 63× magnification with a 4× digital zoom.
